# Effects of Voluntary Running in the Female Mice Lateral Septum on BDNF and Corticotropin-Releasing Factor Receptor 2

**DOI:** 10.1155/2011/932361

**Published:** 2011-09-29

**Authors:** Sofia Gustafsson, Wen Liang, Susanne Hilke

**Affiliations:** ^1^Division of Clinical Chemistry, Department of Clinical and Experimental Medicine, Linköping University, 58183 Linköping, Sweden; ^2^TNO Metabolic Health Research, 2333 Leiden, The Netherlands

## Abstract

Voluntary physical activities are known to modulate anxiety and depressive/like behaviors in both animals and humans. Brain derived neurotrophic factor (BDNF), has been reported to be elevated following exercise. BDNF, as well as type 2 corticotrophin releasing factor receptor (CRFR) 2, has been shown to mediate anxiety-like behavior. In the present study we examined the effects of long-term voluntary exercise on the transcripts for BDNF and CRFR2 in the lateral septum (LS) and for CRF in the central amygdala (CeA) in female mice. Thus, increased activity of CRF in the CeA is associated with anxiety-like behavior. Quantitative RT-PCR was employed to measure levels of mRNA in punch biopsies from LS and CeA. In addition, measurements of the concentration of corticosterone and leptin in plasma were employed. In the LS, we found a three-fold increase of BDNF mRNA (*P* < 0.05) but no significant change in CRFR2 mRNA. No changes in CRF in the amygdala were observed but we found a decrease in the levels of plasma corticosterone. Plasma leptin and the weight of perigonadal fat pads were decreased following exercise. In conclusion, these data show that BDNF gene expression in the LS is influenced by long-term exercise in females but not CRFR2.

## 1. Introduction

Voluntary exercise has been found to mitigate harmful consequences of stress on the brain and to prevent the expression of depression and anxiety-like behavior [1].Increased activity of brain-derived neurotrophic factor (BDNF) signaling is suggested to be an important factor mediating the benefits seen after running.In agreement, stress and depression have been found to decrease BDNF expression [2, 3].

Corticotrophin-releasing factor (CRF) is the major hypothalamic mediator of stress and is also involved in the etiology of depression and anxiety-like behavior [4, 5] and has been found to be downregulated in the mouse hypothalamus following exercise [6, 7]. CRF acts through the seven transmembrane, G-protein-coupled receptor CRF receptor 1 (CRFR1), initiating the release of cortisol in humans and corticosterone in rodents from the adrenal gland [8]. The cloning of a second CRF receptor (CRFR2) [9–11], existing as two primary splice variants (CRFR2*α* and CRFR2*β*), was followed by the discovery of three additional CRF family members, the urocortin 1 (Ucn1) [12], urocortin 2 (Ucn 2) [13, 14], and urocortin 3 (Ucn 3) [15]. In general, there is a limited overlap in the distribution of CRF and the CRF receptor subtypes and of the urocortins, suggesting separate but complementary functional roles [16]. The physiological roles of CRFR2 in the brain are still somewhat elusive, but most reports in the literature suggest involvement in dampening the body's response to stress. Thus, mice deficient in CRFR2 (CRFR2 −/−) exposed to restraint stress show rapid and elevated ACTH levels compared to control animals, and behavioral studies show an increase in anxiety-like behaviors [4], possibly due to increased CRF mRNA levels in the central nucleus of the amygdala. However, studies using agonists and antagonists against CRFR2 indicate that the attenuation of depression and anxiety-like behavior in relation to activated CRFR2 is complex. Thus, the effects has been found to be diverse, site specific, and depending on the stress level and experimental model as well as being different between genders [17].

The lateral septum (LS), a brain area important in the shaping of coping responses to stress and found to modulate the activity in the amygdala [18], exhibits the highest density of CRF2 in the brain [16]. Here, we have studied CRFR2 and BDNF in the female mouse LS using quantitative real-time reverse transcriptase polymerase chain reaction (qRT-PCR) in an exercise paradigm. Brain punch biopsies from young female mice exposed to voluntary exercise and nonexercised age- and sex-matched controls were analyzed for expression levels of transcript for BDNF and CRFR2 in LS. In addition, CRF mRNA was studied in the central nucleus of amygdala (CeA), as this is a key site for the integration of central stress circuits. 

Finally, we monitored the exercise-induced effects on morning corticosterone and leptin levels in plasma as well as the weight of abdominal fat mass.

## 2. Materials and Methods

The experiment was performed in 20 naïve, 5-week-old C57/BL6 female mice (Scanbur, Sollentuna, Sweden). All experimental procedures in animals were approved by the Animal Care and Use Committee at Linköping University and in accordance with the European Communities Council Directive guidelines. The mice were housed individually under standard conditions with free access to water and food under a 12-h light and 12-h dark cycle (lights on at 07:00 am). The experimental groups (*n* = 10) had access to a wheel (*∅*: 13 cm) for three weeks, and the housing of the control group (*n* = 10) remained unchanged. The mice were handled every other day by the examiner to decrease stress. Vaginal smears were used to assess the stage in the estrous cycle and all females were in the estrous phase at the end point of the experiment. The amount of running was on average 8,000 revolutions per day with peaks during the proestrus phase.

For analysis of gene expression by qRT-PCR, the mice were euthanized after three weeks at 9.00 am by means of decapitation. Blood was immediately withdrawn from the right ventricle by heart puncture, collected in EDTA containers (Sarstedt AB, Landskrona, Sweden) and centrifuged at 7,000× g (4°C; 10 min). The brains were dissected out, sliced, and put on ice, and punch biopsies were collected under a microscope using an air-powered punching machine following a microdissection protocol. The biopsies were snap-frozen on dry ice and stored at −70°C. The remaining slices were frozen for staining and anatomical localization of the biopsies. Total RNA was extracted using RNeasy Lipid Tissue Mini Kit (Qiagen, Sollentuna, Sweden)—including DNase treatment—and RNA was reversely transcribed to cDNA (Applied Biosystems, Foster City, Calif, USA). Quality control of RNA extraction was performed on each brain area using the Agilent RNA 6000 Nano Assay Protocol (http://www.agilent.com/chem/labonachip/) according to their protocol.

Real-time RT-PCR was performed on an Applied Biosystems 7900 Fast Real-Time PCR System using TaqMan Fast Universal PCR Master Mix according to the manufacturer's instructions (Applied Biosystems, Stockholm, Sweden). The TaqMan Gene Expression assays used were: BDNF = Mm 01334042-m1; CRFR2 = Mm 00438303-m1; B-actin = Mm 00607939-s1 and GAPDH = Mm 99999915-S1 as endogenous controls. Gene expression was calculated using the ΔΔCt method (Ct = threshold cycle). Each gene was normalized with the corresponding average of B-actin and GAPDH expression in the same animal and expressed as the fold difference in relation to the control group. 

Morning corticosterone was measured by means of an enzyme immunoassay (OC-TEIA), according to the manufacturer's instruction (IDS Nordic, Herlev, Denmark). Assay sensitivity was 0.55 ng/mL. Leptin concentration was measured by means of bead-based xMAP Luminex Technology (Austin, Tex, USA), using a commercially available kit (Electrabox, Tyresö, Sweden) according to the manufactures protocol. Data were acquired as mean fluorescence intensity, collected by the Star Station software program (Applied Cytometry Systems, Dinnington, Sheffield, UK). The detection limit for leptin was 3.0 pg/mL. 

All statistical analyses were performed in Statistica 10. For comparisons between several groups, data were analyzed with ANOVA followed by Student's *t*-test to ascertain which group differed significantly from controls. A value of *P* < 0.05 was considered significant.

## 3. Results and Discussion

**Figure 1:**
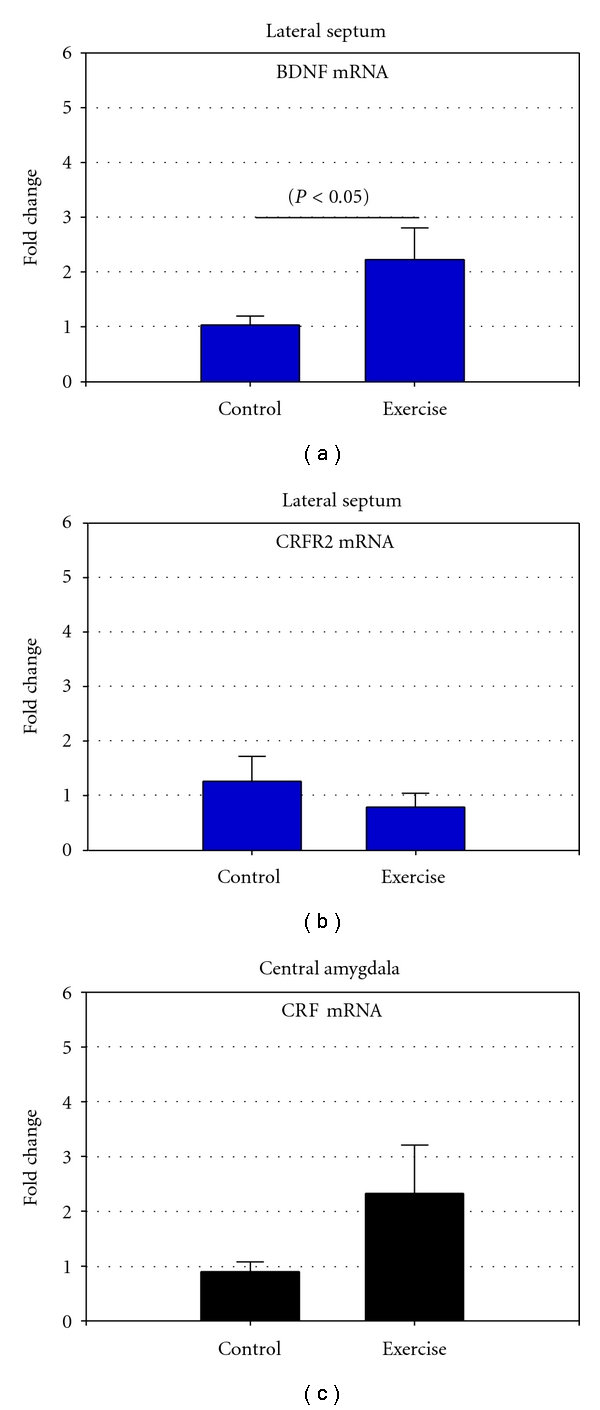
The figure represents the gene expression employed by quantitative real-time PCR (Q-RT-PCR), of brain derived neurotrophic factor (BDNF) (a), corticotrophin-releasing factor receptor 2 (b) in the lateral septum (LS), and corticotrophin releasing factor (CRF) in the central amygdala (CeA) following three weeks voluntary exercise (c). Q-RT-PCR of the full-length BDNF demonstrated a significant, 3-fold upregulation in the LS after three weeks of running compared to controls (*P* < 0.05). No significant changes in CRFR2 (LS) or CRF (CeA) gene expression were observed.

**Figure 2:**
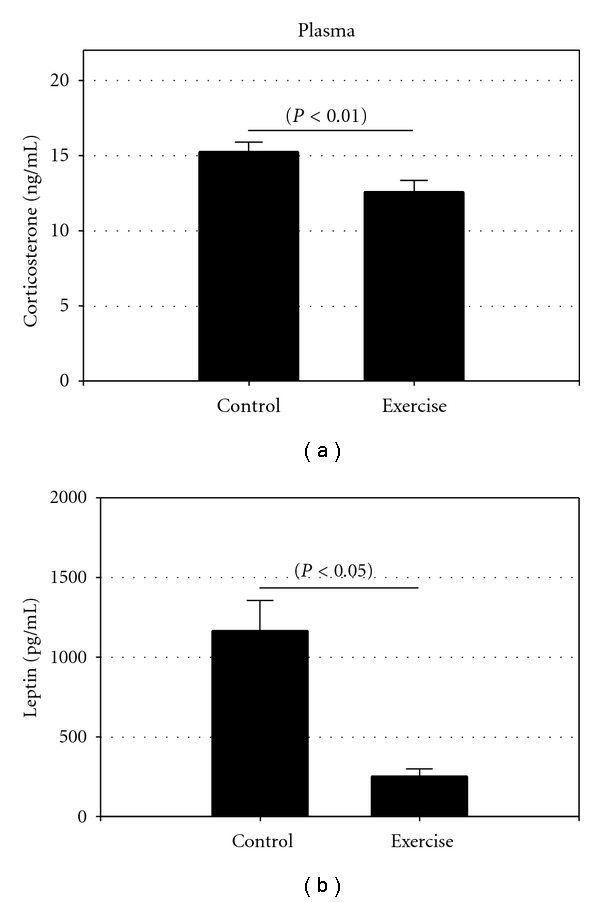
The figure represents plasma levels of corticosterone (a) and leptin (pg/mL) (b) following three weeks voluntary exercise. Corticosterone decreased by 18% in the female runners (*P* < 0.01) and leptin by 60% (*P* < 0.05).

Here, we report a 3-fold increase in BDNF gene-expression levels in the LS following three weeks of voluntary running (*P* < 0.05) (Figure 1). To our knowledge, this is the first demonstration that BDNF mRNA levels in the female LS are markedly increased following long-term exercise. BDNF has a multitude of actions on neurons [19], and dysfunction of this neurotrophin may modulate mood [2]. Exercise has been found to induce BDNF-mediated increase of neurogenesis [20], and increased BDNF gene-expression and protein following acute or long-term exercise results in mood benefits and enhanced memory [3, 21]. However, studies of exercise-induced effects on BDNF in other brain areas and circuits other than hippocampus are limited. In addition, we studied the effect after voluntary exercise on CRFR2, since the LS harbors substantial levels of CRFR2 [16] and since this receptor has been reported to mediate coping responses during the recovery phase after stress [4, 22]. In the present study, CRFR2 gene-expression was, however, not altered after three weeks exercise nor was CeA expression of CRF mRNA although we found a trend towards an increase in CRFR2 (Figure 1). Plasma concentrations of morning corticosterone were decreased by approximately 18% compared to the control group (15,3 ± 1,6 ng/mL; 12,6 ± 2,4 ng/mL, *P* < 0.01) (Figure 2), which may suggests that it is possible that a part of the HPA-axis is inhibited by voluntary long-term exercise. However, this needs to be clarified. We used female mice, since anxiety and depression are twice as common in females, and there is accumulating evidence that the CRF system also outside hypothalamus is differently regulated when comparing female and male rodents [23]. The amount of voluntary running in females is closely correlated to the levels of sex hormones. We observed what others also have found that the average frequency of running where higher during the proestrus phase when there is a peak in estrogen levels [24]. We chose to collect the brains the day at estrus when estrogen levels are low, since it is known that BDNF is regulated by this sex hormone itself at least in the hippocampus. Thus, BDNF-synthesizing neurons express estrogen receptors, and the BDNF gene contains an estrogen responsive element, suggesting possible interactions between estrogen and BDNF regulation [25, 26].

Our working hypothesis was that since acute exercise initiates the activation of the HPA-axis, CRFR2 might be activated to counteract the CRF/CRFR1 system in the amygdala through the LS. Thus, the activation of the CRF system in the amygdala is tightly correlated to stress and anxiety-like behavior [27]. However, neither CRFR2 nor CRF mRNA were changed after three weeks running. Taken together, if CRFR2 and LS are involved in the strategies of stress coping, it seems that long-term voluntary exercise is a situation when LS CRFR2 and CeA CRF are not activated. 

The total body weight of the animals did not change after running. However, we found a decrease of plasma leptin by 60% (*P* < 0.05) (Figure 2), in parallel with a 30% reduction in fat mass (*P* < 0.001). Food intake and water consumption was not measured in the present study, but previous studies of mice using the same experimental model showed no difference in food intake in exercised animals versus controls, and no difference between groups in total body weight [6], similar to what we observed. Leptin is a 16 kDa protein hormone and a product of the obese gene that has been found to be correlated with the lipid content of the cells and plays an important role in regulating food intake and energy expenditure. It has been shown in both humans and mice that weight loss is associated with a decrease in plasma leptin [28]. As already mentioned, we did not observe any changes in total body weight but a decrease in perigonadal fat pads. A decrease in abdominal fat in combination with no weight loss is probably due to an increase in muscle weight after long-term exercise. It is possible that the decrease of plasma leptin in the present study is correlated to the decrease in perigonadal fat. However, further studies are needed, since the mechanisms responsible for regulating leptin expression and protein are complex and not fully understood at least not in females.

In summary, these data show for the first time that long-term voluntary exercise increases BDNF gene expression but not CRFR2 in the LS or CRF in the CeA in young healthy female mice. Although our data show that the BDNF gene is activated by exercise in the LS, the limitation is that we did not measure BDNF protein levels. However, it is possible that exercise may promote LS neuronal survival through increased BDNF, but this needs to be further elucidated. In addition, to what extent this is related to an effect on depressive and anxiety-like behavior remains to be analyzed and would be an intriguing future perspective.

##  Conflict of Interests

The authors declare that there is no conflict of interests related to the study.
